# 

**Published:** 1887-07

**Authors:** 


					﻿Whole Number
CCCI>
LY* 1887.
Number 12.
Volume XXVII.
The BOFFALO
Mhuyudgwud ojfeh iufqehio
ESTABLISHED
IN YEAR 1844.
EDITORS:
THO8, LOTHROP, M. D.
A. Jft. DAVIDSON, !W. I).
ASSOCIATE EDITORS:
FLOYD S. CREGO, M. D.
FRED. R. CAMPBELL, M. D.
CARLTON C. FREDERICK, M. D.
FRANK H. POTTER, M. D.
EDWARD B. ANGELL, M. D., Rochester. N. Y.
Entered at the Post-Office at Buffalo, N. Y., as Second-class Mail Matter.
				

